# Development, Functional Characterization, and Matrix Effectors Dynamics in 3D Spheroids of Triple-Negative Breast Cancer Cells

**DOI:** 10.3390/cells14171351

**Published:** 2025-08-30

**Authors:** Nikolaos E. Koletsis, Sylvia Mangani, Marco Franchi, Zoi Piperigkou, Nikos K. Karamanos

**Affiliations:** 1 Biochemistry, Biochemical Analysis & Matrix Pathobiology Research Group, Laboratory of Biochemistry, Department of Chemistry, University of Patras, 26504 Patras, Greece; nkoletsis@upatras.gr (N.E.K.); sylvia.mangani@upatras.gr (S.M.); zoipip@upatras.gr (Z.P.); 2 Department for Life Quality Studies, University of Bologna, 47921 Rimini, Italy; marco.franchi3@unibo.it

**Keywords:** extracellular matrix, breast cancer, spheroids, 3D breast cancer cell models, estrogen receptor beta, microRNAs, epithelial-to-mesenchymal transition

## Abstract

Breast cancer (BC) remains a leading cause of cancer-related mortality in women. Extracellular matrix (ECM) remodeling is a critical modulator of tumor invasion and metastasis. Three-dimensional (3D) cell culture models have been proposed as advanced systems better mimicking the tumor microenvironment (TME), potentially offering enhanced insights into underlying mechanisms compared to conventional two-dimensional (2D) cultures. This study highlights how BC cells develop metastatic potential and tumor progression independently from ECM contact using advanced 3D spheroid culture models compared to traditional 2D cultures in triple-negative breast cancer (TNBC) cell lines. Spheroids were formed using ultra-low adhesion plates, and their morphological and functional properties were assessed via phase-contrast and scanning electron microscopy (SEM), along with functional assays. Both cell lines formed compact spheroids exhibiting mesenchymal-to-epithelial transition (MET) characteristics. Functional assays showed enhanced cell migration and dissemination of spheroid-derived cancer cells. Gene expression profiling revealed increased expression of ECM remodeling enzymes, cell surface receptors, and adhesion molecules in 3D cultures compared to 2D. MicroRNA analysis highlighted distinct regulatory patterns specifically associated with metastasis and epithelial-to-mesenchymal transition (EMT). These findings demonstrate that 3D spheroid models effectively recapitulate the complexity of TNBC, providing valuable insights into ECM dynamics, epigenetic regulation, and metastatic behavior and potentially guiding improved therapeutic strategies.

## 1. Introduction

Cancer remains one of the leading causes of death worldwide, with metastasis accounting for over 90% of fatalities [1,2]. Tumor progression relies on dynamic interactions with the tumor microenvironment (TME), particularly through extracellular matrix (ECM) remodeling, which is strongly associated with an increased matrix metalloproteinase (MMP) activity [3,4]. Changes of the ECM in TME promote sustained angiogenesis, immune evasion, and other critical cancer hallmarks [5,6].

BC is the most frequently diagnosed malignancy in women, accounting for approximately 30% of all new female cancer diagnoses, posing significant therapeutic challenges, especially in the TNBC subtype [7]. TNBC lacks expression of estrogen receptor alpha (ERα), progesterone receptor (PR), and human epidermal growth factor receptor 2 (HER2), is typically more aggressive, and currently lacks targeted therapies. BC arises predominantly from epithelial cells lining terminal duct lobular units and progresses through stages of in situ to invasive carcinoma [8]. Incidence is influenced by genetic predispositions, hormonal exposure, lifestyle choices, and environmental factors [9]. Although early detection and advances in targeted therapies have improved survival outcomes in other subtypes, our understanding of the mechanisms of growth, invasion, and metastatic potential of the highly aggressive TNBC tumors is limited.

ERs, particularly ERα and ERβ, significantly influence BC progression, with ERα being a key driver in hormone-responsive BC subtypes [10,11]. In TNBC, where ERα is absent, ERβ is expressed at very low or undetectable levels, and its suppression has been associated with altered ECM interactions, dysregulated integrin-FAK signaling, and enhanced migratory and invasive behavior [11,12]. ERs mediate estrogen effects through both genomic and non-genomic signaling pathways, influencing cell proliferation, survival, and ECM remodeling [13]. Emerging evidence indicates that ECM properties—such as collagen I enrichment and increased stiffness—can enhance ER signaling. These mechanical cues are transduced through integrin-mediated FAK pathways, ultimately amplifying ERα activity and downstream gene expression [14]. Although TNBC tumors are characterized as ERα-negative, the ERβ has been shown to play a crucial role in their properties [11,15].

The ECM undergoes extensive remodeling during premetastatic niche formation, invasion, and metastasis. In breast cancer, ECM composition becomes enriched in fibrillar collagens, fibronectin, and hyaluronan, while increased crosslinking leads to matrix stiffening. These changes enhance mechanotransduction, support tumor cell survival, and promote epithelial-to-mesenchymal transition (EMT). Key mediators—including cell surface receptors (e.g., EGFR and SDC4) and transmembrane syndecans (SDCs)—play crucial roles in several cellular functions such as cell migration, adhesion, and signal transduction and have been related to tumor growth and progression, particularly in aggressive subtypes such as TNBC. Proteolytic enzymes, including MMPs and urokinase-type plasminogen activator (uPA), are pivotal for degrading ECM substrates and facilitating invasion [16]. These matrix effectors contribute to the formation of a stiff, pro-invasive microenvironment, which activates intracellular signaling pathways, enhancing tumor cell dissemination [4,11,17].

Traditional two-dimensional (2D) monolayer cultures have provided valuable insights into BC biology; however, they fail to replicate the spatial architecture, mechanical cues, and complex cell–ECM interactions of the TME, limiting their translational relevance [5]. In contrast, three-dimensional (3D) culture systems, such as multicellular tumor spheroids, better mimic the organization, cell–matrix interactions, and heterogeneity found in tumors, including ECM remodeling, signaling gradients, and cellular heterogeneity [18,19]. These features make spheroids a more physiologically relevant model for studying ECM-associated processes—such as MMP activity, cell receptor regulation, and SDC-mediated signaling—as well as for evaluating therapeutic responses. Evaluation of the functional properties of TNBC cells and analyzing the expression of key matrix remodeling and signaling-mediating molecules in 3D culture conditions in comparison with the traditional 2D cell cultures will improve our understanding of the matrix-driven mechanisms in the development, growth, and progression of TNBC.

MicroRNAs (miRNAs) are also emerging as important regulators of EMT, ECM remodeling, and metastasis, a role that has also been demonstrated by our group [20,21]. Differential expression of miRNAs in 3D cultures reflects changes in the tumor’s transcriptional and post-transcriptional regulatory networks, largely driven by the 3D cellular architecture and matrix-mediated cues [22]. This study leverages a 2D vs. 3D comparative framework to dissect how matrix-related gene and miRNA expression profiles shift in TNBC, providing deeper insights into the mechanistic links between ECM remodeling and metastatic potential [19,23]. For this purpose, we developed and characterized 3D BC cell-derived spheroid models from the aggressive ERβ-very low/negative TNBC cell lines (shERβ MDA-MB-231 and Hs578T), evaluating the functional and molecular consequences on ECM remodeling and mesenchymal signaling.

The suppression of ERβ in MDA-MB-231 cells was employed to mimic the loss of ERβ observed in more invasive TNBC subtypes and to investigate its regulatory role on ECM-associated genes, particularly matrix metalloproteinases (MMPs), growth factor receptors, and adhesion molecules. ERβ is increasingly recognized as a key player in tumor progression in breast cancer, and its downregulation has been linked to alterations in cell morphology, invasiveness, EMT, and poor prognosis. Consistent with established approaches for ERβ knockdown validation in breast cancer cell lines, such as qRT-PCR and Western blot confirmation, we confirmed efficient suppression of ERβ expression in ERβ-modified MDA-MB-231 cells prior to downstream assays [15]. This ensured that subsequent analyses reflected ERβ-dependent mechanisms of the expression of matrix effectors in a clinically relevant TNBC context. Therefore, the use of shERβ-modified MDA-MB-231 cells allowed us to probe the ERβ-dependent mechanisms of the matrix gene expression profiles in a clinically relevant TNBC context. Results will elucidate matrix effectors’ dynamics in 3D conditions, supporting spheroids as robust models for mechanistic studies and therapeutic testing.

## 2. Materials and Methods

### 2.1. Cell Cultures and Reagents

shERβ MDA-MB-231 (MDA-MB-231-derived ERβ-suppressed cells) and Hs578T, TNBC cell lines were both incubated in monolayer cultures at 37 °C in an atmosphere of 5% CO_2_ and 95% humidified atmospheric air. The cell culture medium utilized was Dulbecco’s modified Eagle’s medium (DMEM; LM-D1110/500, Biosera, Cholet, France), enriched with 10% fetal bovine serum (FBS; FB-1000, Biosera, Cholet, France), as well as with antimicrobial agents (100 IU/mL penicillin, 100 μg/mL streptomycin, 10 μg/mL gentamycin sulfate, and 2.5 μg/mL amphotericin B, Biosera, Cholet, France), and 2 mM L-glutamine (Biosera, Cholet, France). The cells were recultured after using trypsin-EDTA 1X diluted in PBS (LM-T1706; Biosera, Cholet, France) at 80–85% cell confluency.

### 2.2. 3D Cell Cultures and Spheroids Development

BC cell-derived 3D spheroids were formed by incubating cancer cells in 96-well round-bottom plates with ultra-low adhesive properties (SPL Life Sciences, Pocheon-si, Republic of Korea). Specifically, shERβ MDA-MB-231 and Hs578T were seeded at a density of 15,000 cells per well and incubated for 72 h in complete medium (DMEM 10% FBS), without any medium change, allowing for spheroid development and formation. Spheroid development was monitored through phase-contrast microscopy. Subsequently, the medium was discarded, and cells were further cultured following a 16–20 h starvation period (0% FBS). This step was implemented to minimize serum-induced signaling and synchronize cellular states prior to downstream analyses. The duration of starvation was selected based on prior literature and preliminary optimization experiments, ensuring sufficient pathway quiescence without compromising cell viability or spheroid integrity. Subsequent analyses included morphological characterization of the spheroids under a phase contrast microscope (OLYMPUS CKX41, OLYMPUS, Center Valley, PA, USA) and SEM. The spheroids were then either collected for total RNA extraction or transferred to standard flat-bottom well plates to examine their spreading and migratory properties accordingly.

### 2.3. Scanning Electron Microscopy

To analyze the morphological properties of 3D BC spheroids, SEM imaging was utilized. The development of BC cell-derived spheroids (5000 cells/spheroid) was followed by washes with a phosphate buffer solution at 0.2 M and pH 7.4. Afterwards, spheroids were fixed in Karnovsky’s fixation solution 4% (*w*/*v*) paraformaldehyde, 5% (*v*/*v*) glutaraldehyde, 0.04 M phosphate buffer solution for 1 h at RT to preserve their structural integrity. Subsequently, spheroids were washed with 0.1% cacodylate buffer and were post-fixed in 1% OsO_4_ in cacodylate buffer for 20 min. Afterwards, they were dehydrated with increasing concentrations of EtOH and finally with HMDS. The samples were coated with a 5 nm palladium gold film (Emitech 550 sputter-coater, Richmond Scientific, Chorley, UK). Finally, observation of the 3D spheroids was performed in a SEM microscope (Philips 515, Eindhoven, The Netherlands) in the secondary electron mode.

### 2.4. Spheroid Dissemination Assay

After 72 h of spheroid culture, a 4 h starvation period was implemented. Subsequently, the spheroids were transferred to a regular, flat-bottom 96-well plate containing 1 spheroid per well in 5% FBS-enriched DMEM to assess cancer cell dissemination. Representative photos were captured using a phase-contrast microscope equipped with a digital camera after 24 and 48 h of cancer cell spreading.

### 2.5. Wound Healing Assay

To evaluate the monolayer cell culture migratory capacity, shERβ MDA-MB-231 and Hs578T cells were seeded into 48-well flat-bottom plates at a density of 15,000 cells per well and incubated in 10% FBS-enriched DMEM medium until the monolayer was formed after 24–48 h. The medium was then replaced with serum-free medium (0% FBS), and the cells were serum-starved overnight. The following day, a straight-line wound was engraved in the cell layer using a sterile 10 μL pipette tip, and each well was washed 2–3 times with serum-free medium in order to remove detached cells. Serum-free medium containing the cytostatic agent cytarabine (10μΜ) was added to eradicate the possibility of contributing to cell proliferation. After 40 min of incubation with cytarabine at 37 °C in an atmosphere of 5% CO_2_ and 95% atmospheric air, representative images (0 h) were captured using a phase-contrast microscope equipped with a digital camera. Additional images were taken at 24 and 48 h, and cancer cell migration was assessed by quantifying the wound surface area (ImageJ 1.50b Launcher Symmetry Software, LOCI; University of Wisconsin, Madison, WI, USA). To assess the migratory capacity of spheroid-derived cells, 5 spheroids from each cell line (cultured for 72 h and serum-starved for 4 h) were transferred into a well of a 48-well plate (DMEM 5% FBS) and allowed to adhere and spread until they reached confluency. After 3 days for both cell lines, the wound healing assay was conducted in the spheroid-derived cells, as described.

### 2.6. Spheroid Growth

The BC 3D spheroids were formed by incubating the cancer cells in 96-well round-bottom plates with ultra-low adhesive properties. Specifically, shERβ MDA-MB-231 and Hs578T were seeded at a density of 15,000 cells per well and incubated for 48 h in DMEM, enriched with 10% FBS, as well as with antimicrobial agents (100 IU/mL penicillin, 100 μg/mL streptomycin, 10 μg/mL gentamycin sulfate, and 2.5 μg/mL amphotericin B) and 2 mM L-glutamine without any medium change, allowing for spheroid development and formation. After successful formation, their growth in size was monitored through phase-contrast microscopy every 24 h. Subsequently, the medium was renewed, with two half-medium changes (50 μL out of the total 100 μL) every 48 h, and cells were further cultured for a total of 10 days, while the cells were monitored every 24 h.

### 2.7. RNA Extraction, cDNA Synthesis, and Real-Time qPCR

For total RNA isolation from 2D cell cultures, shERβ MDA-MB-231 and Hs578T cells were seeded at a density of 15,000 cells per well in 10% FBS-enriched DMEM medium for 72 h until reaching 80–85% confluence. Subsequently, the medium was replaced with serum-free medium, and cells were serum-starved for 16–20 h. To harvest the cells, trypsin-EDTA 1X in PBS, followed by two washes with cold PBS 1X, each performed by centrifugation at 2400 rpm for 3 min, was utilized. Total RNA isolation was performed using NucleoSpin^®^ RNA II Kit (MACHEREY-NAGEL, Dueren, Germany), which includes treatment with recombinant DNase (rDNase) to eliminate potential genomic DNA contamination, following the manufacturer’s instructions. For total RNA isolation from 3D cell cultures, BC cell-derived spheroids were cultured for 72 h and starved for 16–20 h. This step was implemented to minimize serum-induced signaling and synchronize cellular states prior to downstream analyses. The duration of starvation was selected based on prior literature and preliminary optimization experiments, ensuring sufficient pathway quiescence without compromising cell viability or spheroid integrity. A total of 90 spheroids, each seeded from 15,000 cells, were collected for RNA extraction. Afterwards, spheroids were collected, and their total RNA was isolated, as described above, with the only differentiation being that Trypsin EDTA solution was not used; instead, spheroids were harvested with a 1000 μL pipette tip. Quantification of the total isolated RNA was performed by measuring its absorbance at 260 nm via a photometer, and its purity was ensured by evaluating the 260/280 nm and 260/230 nm ratios of all RNA extracts. Total RNA from both 2D and 3D cultures was reverse-transcribed, using the PrimeScript 1st strand cDNA synthesis kit (Takara Bio Inc., Goteborg, Sweden), perfect real-time. Real-time qPCR analysis was conducted according to manufacturer’s guidelines, while the Rotor Gene Q (Qiagen, Germantown, MD, USA) was utilized for the amplification process. To further ensure the absence of DNA contamination, non-template controls (NTCs) were included in all qPCR reactions and melt curve analyses were performed to confirm the specificity of the amplified products. A NTC sample, containing mastermix reagents and primers, but replacing cDNA with PCR-grade water, was included as negative control. The reactions were performed in triplicate, with a standard curve for each primer pair to validate the assay. Additionally, we performed a melting curve analysis to detect the SYBR Green Materials and Methods-based target amplicon. The fluorescence threshold, set above the background, was used to determine the threshold cycle (Ct) number, representing the point of product accumulation during the early logarithmic phase of amplification. Subsequently, we calculated the relative expression of different gene transcripts by the ΔΔCt method, where the Ct of each gene of interest was normalized to the Ct of the housekeeping gene ACTB. Among other normalizers (e.g., GAPDH), ACTB was selected as the reference gene for normalization due to its stable expression across both 2D and 3D culture conditions in our experimental system. Fold changes (arbitrary units) were determined as 2^−ΔΔCt^. Detailed information about the target genes, as well as the utilized primers, is listed in Table 1. The genes included in Table 1 were selected based on their established roles in extracellular matrix remodeling (e.g., MMP2, MMP7, MMP9, and uPA/PLAU), cell signaling pathways implicated in tumor progression (EGFR, IGF1R), and cell adhesion or EMT plasticity (e.g., SDC4, and F11R). These genes are known to be dysregulated in breast cancer and play a central role in processes such as invasion, metastasis, and resistance to therapy. Their relevance was further confirmed by Kaplan–Meier survival analyses and differential expression patterns in our 2D vs. 3D models.

### 2.8. miRNA Screening

For total RNA isolation from 2D cell cultures, shERβ MDA-MB-231 and Hs578T cells were seeded at a density of 15,000 cells per well in complete medium for 72 h until reaching 70–80% confluence. Subsequently, the medium was changed to serum-free, and cells were serum-starved for 16–20 h. This step was implemented to minimize serum-induced signaling and synchronize cellular states prior to downstream analyses. The duration of starvation was selected based on prior literature and preliminary optimization experiments, ensuring sufficient pathway quiescence without compromising cell viability or spheroid integrity. To harvest the cells, trypsin-EDTA 1X in PBS, followed by two washes with cold PBS 1X, each performed by centrifugation at 2400 rpm for 3 min, was utilized. Total RNA isolation was performed using NucleoSpin^®^ RNA II Kit. For total RNA isolation from 3D cell cultures, 90 spheroids were used for RNA extraction, each initially seeded with 15,000 cells per well. These were cultured for 72 h and starved for 16–20 h. Afterwards, spheroids were collected, and their total RNA was isolated, as described above, with the only differentiation being that trypsin EDTA solution was not used; instead, spheroids were harvested with a 1000 μL pipette tip. Quantification of the total isolated RNA was performed by measuring its absorbance at 260 nm using a photometer, and its purity was ensured by evaluating the 260/280 nm and 260/230 nm ratios of the RNA extracts. Target miRNAs from 2D and 3D cultures were reverse-transcribed, Mir-X™ miRNA FirstStrand Synthesis (Takara Bio Inc., Goteborg, Sweden), and TB Green^®^ qRT-PCR (Takara Bio Inc., Goteborg, Sweden). Real-time qPCR analysis was conducted according to manufacturer’s guidelines with Rotor Gene Q (Qiagen, Germantown, MD, USA) for the amplification process. A NTC sample, containing all mastermix reagents and primers, but replacing cDNA with PCR-grade water, was included as negative control. All reactions were performed in triplicate, and a standard curve was included for each primer pair to validate the assay. The fluorescence threshold, set above the background, was used to determine the threshold cycle (Ct) number, representing the point of product accumulation during the early logarithmic phase of amplification. The relative expression of different miRNAs was calculated by the ΔΔCt method, where the Ct of each gene of interest was normalized to the Ct of the small 18S rRNA gene. Among 18S rRNA, RNU44, and U6 snRNA normalizers, 18S rRNA was selected as the reference gene for miRNA normalization due to its stable expression across both 2D and 3D culture conditions in our experimental system. Fold changes (arbitrary units) were determined as 2^−ΔΔCt^. Detailed information about the target miRNAs is listed in Table 2. Regarding miRNA selection (Table 2), the included mature miRNAs (e.g., miR-10b, miR-145, miR-200b, and let-7d) are widely recognized as key regulators of EMT and breast cancer progression. Specifically, miR-10b and miR-200b are pro-mesenchymal and epithelial-associated regulators, respectively, while miR-145 and let-7d are involved in suppressing stemness and promoting differentiation. These miRNAs were selected to explore the regulatory axis linking miRNA networks with the expression of EMT/matrix-associated genes in both 2D and 3D culture models.

### 2.9. Bioinformatic Tools Describe

#### 2.9.1. Kaplan–Meier Plotter

Kaplan–Meier Plotter (https://kmplot.com/analysis/, accessed on 25 May 2025) is an online survival analysis tool designed to assess the prognostic significance of gene expression in various cancers, including BC. By integrating transcriptomic data from various public sources, such as TCGA, GEO, and EGA, it enables researchers to generate survival plots based on the expression levels of single genes or gene combinations. Users can stratify patients by median, tertiles, or quartiles of expression and assess outcomes such as overall survival, relapse-free survival (RFS), or distant metastasis-free survival (DMFS). This facilitates the identification of candidate biomarkers associated with patient prognosis and supports hypothesis-driven exploration of gene function in clinical contexts [24].

In this study, we queried several genes of interest (e.g., EGFR, IGF1R, SYND4, and PLAU). The “Breast Cancer” dataset was selected. Patient stratification was set to median split (high vs. low expression), and the endpoint analyzed was RFS. The resulting plots provided hazard ratios (HRs), confidence intervals (CIs), and log-rank *p*-values to evaluate the prognostic relevance of gene expression levels.

#### 2.9.2. Human Protein Atlas

The Human Protein Atlas (HPA, https://www.proteinatlas.org/, accessed on 25 May 2025) is a comprehensive open-access resource that maps protein expression and localization across a wide range of human tissues, cell types, and organ systems. It integrates transcriptomics, antibody-based imaging, and mass spectrometry data to build a multiscale view of protein biology. The platform is divided into specific modules, including the Tissue Atlas, Cell Atlas, and Pathology Atlas, each offering insight into normal physiology or disease-associated expression patterns. Of particular relevance is the Pathology Atlas, which links protein expression to cancer types and patient survival data, aiding in the identification of clinically relevant targets and biomarkers [25].

We searched for the expression profiles of the same genes in the “Cell Line” and “Pathology” sections of the HPA. Specifically, we retrieved nTPM values for MDA-MB-231 and Hs578T breast cancer cell lines. The expression data provided complementary evidence for gene relevance in triple-negative breast cancer models. These findings supported our gene selection for further validation.

### 2.10. Statistical Analysis

The data are presented as a mean +/− standard deviation (SD) from experiments conducted in triplicate. Statistical significance was assessed using one-way analysis of variance (ANOVA), followed by Tukey’s post hoc test to identify differentiations between the two groups (2D and 3D cell models). A *p*-value of ≤0.05 was considered statistically significant, with asterisks (*) indicating significant differences in 3D cultured cells relative to their 2D counterparts. All statistical analyses and graphs were made using GraphPad Prism version 8.0.1 (GraphPad Software, Boston, MA, USA).

## 3. Results

### 3.1. Morphology and Functional Characteristics

**Figure 1:**
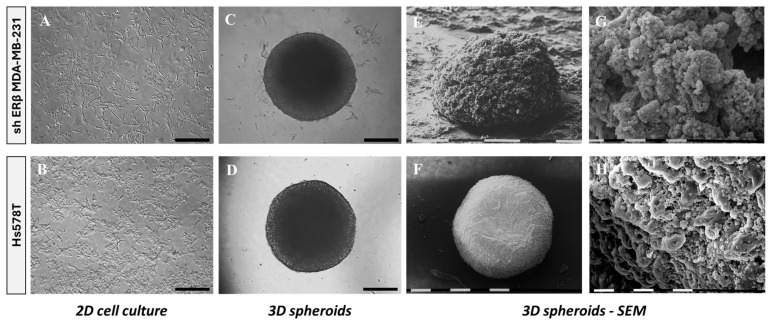
Morphological comparison of shERβ MDA-MB-231 and Hs578T BC cell lines in 2D monolayers and 3D spheroid cultures using phase-contrast and scanning electron microscopy (SEM). (**A**,**B**) Phase-contrast images of cells cultured in 2D show characteristic mesenchymal morphology with elongated spindle-like shapes for both cell lines. Black bar, 200 μm. (**C**,**D**) Phase-contrast images of intact 3D spheroids formed by each cell line demonstrate differences in compactness and edge definition. Black bar, 200 μm. (**E**,**F**) SEM images reveal ultrastructural features of the spheroids, with shERβ MDA-MB-231 forming loosely packed, irregular aggregates, whereas Hs578T spheroids appear denser and more spherical. White/black bar, 0.1 mm. (**G**,**H**) Higher-magnification SEM images highlight surface architecture, showing increased porosity and rough texture in shERβ MDA-MB-231 spheroids, compared to the more compact and smooth appearance of Hs578T spheroids. White/black bar, 10 μm.

To evaluate cancer cell properties and tumor growth in vitro, we developed 3D TNBC-derived spheroids. To this end, shERβ MDA-MB-231 and Hs578T cells were cultured for 72 h in 96-well round-bottom ultra-low adhesion plates. The morphological organization was observed using conventional phase-contrast microscopy and SEM. shERβ MDA-MB-231 cells were selected over the parental cell line (MDA-MB-231) to investigate the specific impact of ERβ suppression on tumor cell morphology and behavior, as ERβ has been implicated in modulating epithelial–mesenchymal plasticity and invasiveness in MDA-MB-231 cells. Phase-contrast images of cells cultured in 2D conditions revealed an epithelial-like morphology in the case of shERβ MDA-MB-231 cells, while maintaining mesenchymal traits, characteristic of their precursors. Hs578T cells are observed in characteristic mesenchymal morphology with elongated spindle-like shapes (Figure 1A,B), consistent with the gene expression analysis. It is worth noticing that both cell lines exhibited invasive characteristics and formed dense globular structured spheroids, reflecting an epithelial-like morphology despite their mesenchymal phenotype, potentially indicative of a mesenchymal-to-epithelial transition (MET).

Intact 3D spheroids formed by each cell line demonstrate slight differences in compactness and edge definition (Figure 1C,D). SEM imaging further illustrated these differences, revealing that Hs578T spheroids appeared denser as compared with shERβ MDA-MB-231 cells (Figure 1E,F). High-magnification SEM images highlighted distinct surface architectures, indicating increased porosity and rough texture in shERβ MDA-MB-231 spheroids compared to the compact and smooth appearance of Hs578T spheroids (Figure 1G,H). These structural differences suggest variations in nutrient penetration potential to the intracellular space and spheroid core.

#### 3.1.1. Spheroid Growth Rate

**Figure 2:**
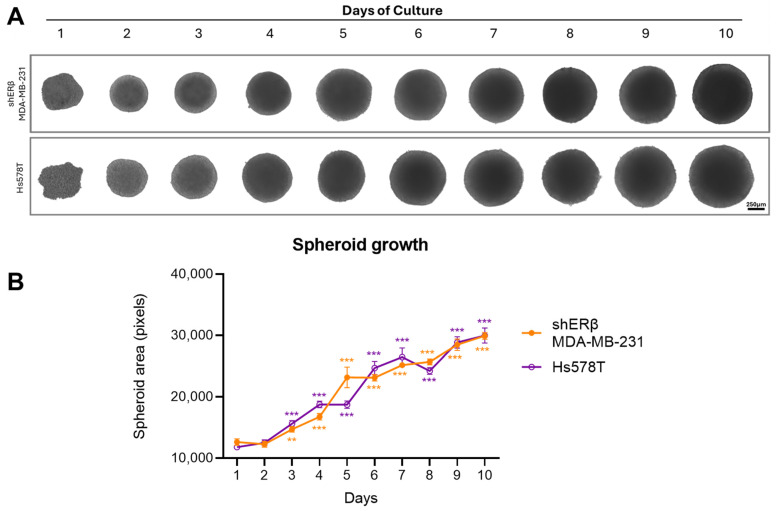
Morphology and growth curve of spheroids obtained by shERβ MDA-MB-231 and Hs578T BC cell lines. The BC 3D spheroids were formed by incubating the cancer cells in 96-well round-bottom plates with ultra-low adhesive properties at a density of 10,000 cells/well. Cells were incubated for 72 h in complete medium without any medium change at 37 °C in an atmosphere of 5% CO_2_ and 95% air, allowing for spheroid development. Subsequently, half medium changes followed every 2 days to support further spheroid growth. (**A**) Representative images showing spheroid growth for 10 days (scale bar, 250 μm). (**B**) Quantitative graph showing area of spheroids for 10 days. Data are expressed as mean ± SD of one experiment. Asterisks (** *p* < 0.01), (*** *p* < 0.001) indicate statistical significance between culture days as compared to day 1 (n = 3).

Spheroid formation and growth dynamics were assessed over a 10-day period to evaluate the self-assembly and expansion capacity of the two BC cell lines, shERβ MDA-MB-231 and Hs578T. As shown in Figure 2, both cell lines successfully formed compact, rounded spheroids, which progressively increased in size over time. Quantitative analysis revealed that while both lines exhibited a significant increase in spheroid area compared to day 1, shERβ MDA-MB-231 spheroids demonstrated a slightly faster and more consistent growth trend compared to Hs578T. These differences were particularly evident between days 5 and 8, as shown by the statistical significance in the corresponding graph. The ability of both cell types to maintain spheroid integrity and expansion supports their suitability for further functional analyses under 3D conditions.

#### 3.1.2. Spreading of Spheroids—Mimicking Initial Steps of Cancer Cell Dissemination

**Figure 3:**
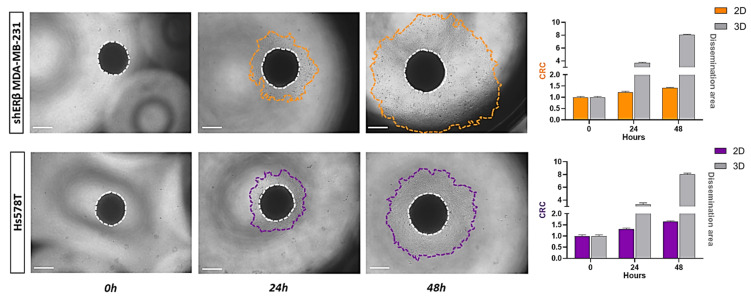
Quantification of cell spreading and dissemination from spheroids of shERβ MDA-MB-231 and Hs578T BC cell lines. Representative images show the initial spheroid at time 0 and progressive outward spreading at subsequent time points, with the boundary of the disseminated cells marked (orange for shERβ MDA-MB-231 and purple for Hs578T). The central core is outlined with a white dashed line. Bar graphs to the right display the expansion of the dissemination area over time, illustrating that spheroids derived from both cell lines exhibit extensive spreading compared. Data represent the mean ± SD from three independent experiments. Areas were calculated utilizing ImageJ analysis. (scale bar, 200 μm).

Following the MET process of 2D cell cultures into 3D spheroids, the spheroids were transferred from 96-well round-bottom ultra-low adhesion plates to standard 96-well ones. The representative phase-contrast images (Figure 3) depict that during spheroid dissemination; the cells tend to revert to their original 2D morphology and characteristics. Marker expression analysis supports the following observations: In shERβ MDA-MB-231 cells, which display an epithelial-like morphology and baseline E-cadherin (CDH1) expression, both *CDH1* and *SLUG* remained low and unchanged during the 3D→2D transition, consistent with maintenance of their epithelial-like phenotype. In contrast, Hs578T cells, which are intrinsically triple-negative with low *CDH1* and high *SLUG* expression in 2D, showed an epithelialization profile in 3D spheroids (CDH1 increased from 1 to 1.66; *SLUG* decreased from 1 to 0.23). Upon dissemination back to 2D, *CDH1* expression declined (1.66→1), while *SLUG* expression rose (0.23→1), indicating a mesenchymal reversion. The phenotypic alteration is present in both cell lines, which shift from the epithelial-like morphology observed in the 3D state to their regular mesenchymal-like 2D state. As observed utilizing the ImageJ tool, the dissemination area of both cell lines expands significantly. Both shERβ MDA-MB-231 and Hs578T spheroids demonstrated active dissemination, although differences in the extent and patterns of cell migration were observed, reflecting the inherent invasive capabilities and differential ECM interactions between the two cell lines. This phenotypic reversion and differential dissemination reflect the dynamic plasticity of TNBC cells and their capacity to adapt to different conditions. In particular, the loss of 3D spheroid cohesion and acquisition of migratory behavior is consistent with the altered expression of ECM-degrading enzymes and adhesion molecules, as supported by our gene expression data. These findings highlight how TNBC cells may reprogram the gene expression of matrix-related effectors in response to environmental changes, recapitulating key steps of early invasion and metastatic seeding.

#### 3.1.3. Wound Healing Capacity of Spheroid-Derived Cells

**Figure 4:**
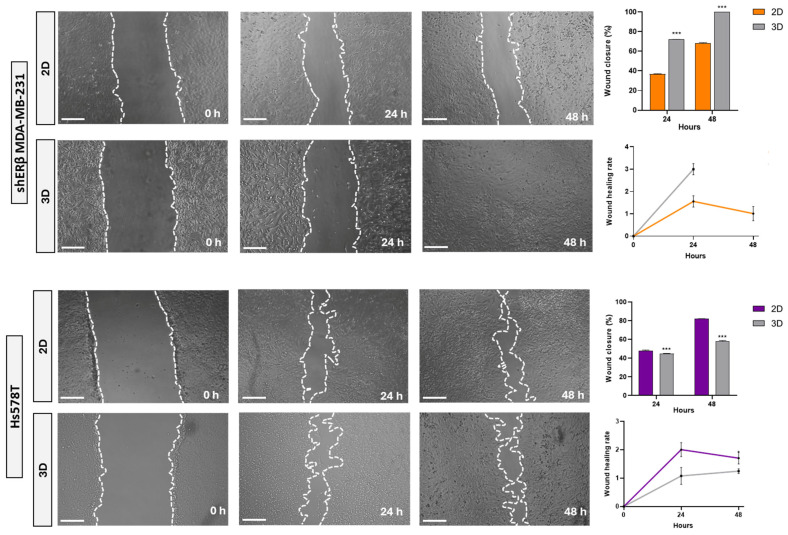
Comparative analysis of migratory capacity in shERβ MDA-MB-231 and Hs578T BC cell lines under 2D and 3D culture conditions using a wound healing assay. Representative phase-contrast images depict wound closure at 0, 24, and 48 h. The white dashed lines indicate the wound edges. Bar graphs and line plots on the right quantify the percentage of wound closure over time for each condition. Orange bars and lines correspond to shERβ MDA-MB-231, while purple indicates Hs578T. Data are presented as mean ± SD from three independent experiments. Statistical significance between time points and culture conditions is denoted by (***) *p*  <  0.001 (scale bar, 500 μm).

The migratory potential of cells derived from spheroids compared to those from 2D monolayers was evaluated using wound-healing assays. The disseminated spheroid-derived cells demonstrated significantly faster migratory capacity compared to their 2D counterparts in the case of the shERβ MDA-MB-231 cell line, which exhibits a higher wound healing rate after 24 h and a complete wound closure at the 48 h mark. Notably, the wound closure in spheroid-derived cells is almost two-fold compared to their 2D counterparts. On the contrary, spheroid-derived cells’ rate and closure percentage seem not to be positively affected in the case of Hs578T. These differentiations with respect to the different rates and wound closure percentages in both states at the same time frames are shown in the corresponding graphs (Figure 4).

### 3.2. Evaluation of the Expression of Key Matrix Effectors in 3D Spheroids

**Figure 5:**
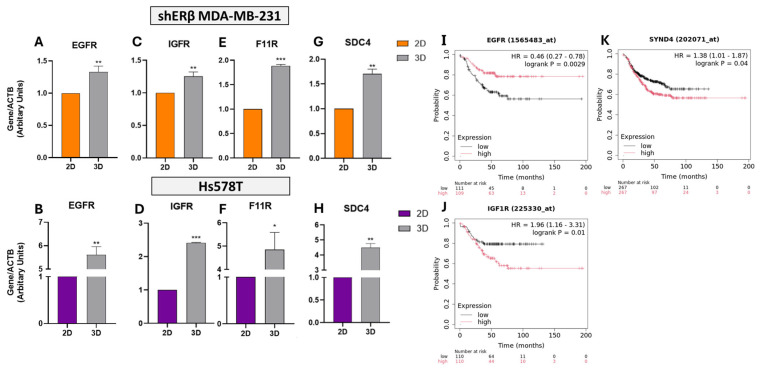
Gene expression analysis of *EGFR*, *IGF1R*, *F11R*, and *SDC4* in shERβ MDA-MB-231 and Hs578T BC cell lines cultured in 2D monolayers and 3D spheroids. (**A**–**H**) Real-time qPCR results show that *EGFR*, *IGFR* (*IGF1R*), *F11R* (*JAM-A*), and *SDC4* are significantly upregulated under 3D culture conditions compared to 2D in both cell lines. Orange bars represent shERβ MDA-MB-231, while purple bars represent Hs578T cells. Bars indicate mean ± SD from triplicate experiments. Each bar indicates the mean ± SD from triplicate experiments. Asterisks denote statistically significant differences between 2D and 3D conditions: (*) *p*  <  0.05, (**) *p*  <  0.01, and (***) *p*  <  0.001, as determined by Student’s *t*-test. (**I**–**K**) Kaplan–Meier survival plots from TNBC patient datasets show that high *EGFR* expression is significantly associated with improved relapse-free survival (RFS) (HR = 0.46, *p* = 0.0029), while *F11R* (*JAM-A*) is not significantly associated with outcome (HR = 0.81, *p* = 0.17). In contrast, *IGF1R* expression correlates with worse RFS (*IGF1R*: HR = 1.96, *p* = 0.01), highlighting its potential prognostic relevance.

To gain mechanistic insight into how 3D architecture alters oncogenic signaling, we assessed the expression of selected ECM- and receptor-related genes in BC spheroids and correlated them with patient survival. Receptor gene expression analysis revealed that culturing BC cells in 3D spheroid conditions significantly altered the expression of key growth-related receptors compared to 2D monolayers (Figure 5).

Real-time q-PCR analysis revealed that *EGFR* expression was significantly upregulated in 3D cultures, showing a 1.4-fold increase in shERβ MDA-MB-231 cells (*p* < 0.01) and a 5.7-fold increase in Hs578T cells (*p* < 0.01) compared to 2D monolayers (Figure 5A,B). Corresponding transcriptomic data from the HPA indicate high basal expression levels of *EGFR*, with 66.1 TPM in MDA-MB-231 and 42 TPM in Hs578T cells, supporting its relevance across models. Kaplan–Meier survival analysis further demonstrated that high *EGFR* expression correlates with improved RFS in TNBC patients (HR = 0.46, *p* = 0.0029) (Figure 5I), suggesting a potential protective role in this subtype.

Similarly, *IGF1R* expression was significantly elevated under 3D culture, with a 1.2-fold increase in shERβ MDA-MB-231 (*p* < 0.01) and a 2.4-fold increase in Hs578T cells (*p* < 0.001) (Figure 5C,D). In clinical datasets, high *IGF1R* expression was associated with significantly poorer RFS (HR = 1.96, *p* = 0.01) in TNBC patients (Figure 5J), indicating a possible context-dependent oncogenic function.

The tight junction-associated molecule *F11R* (JAM-A), a regulator of epithelial polarity, was also upregulated in 3D conditions (1.8-fold in shERβ, *p* < 0.001; 4.9-fold in Hs578T, *p* < 0.05) (Figure 5E,F). Despite this marked induction, F11R expression did not significantly affect patient outcomes (HR = 0.81, *p* = 0.17), suggesting limited prognostic value in this context.

Lastly, the expression of *SDC4*, a transmembrane heparan sulfate PG involved in cell–ECM signaling, was significantly increased in 3D spheroids (1.7-fold in shERβ, *p* < 0.01; 4.5-fold in Hs578T, *p* < 0.01) (Figure 5G,H). High expression of *SYND4* (the gene encoding SDC4) was associated with reduced RFS (HR = 1.38, *p* = 0.04) in TNBC patients (Figure 5K), highlighting its potential role in promoting tumor progression through matrix-mediated mechanisms.

**Figure 6:**
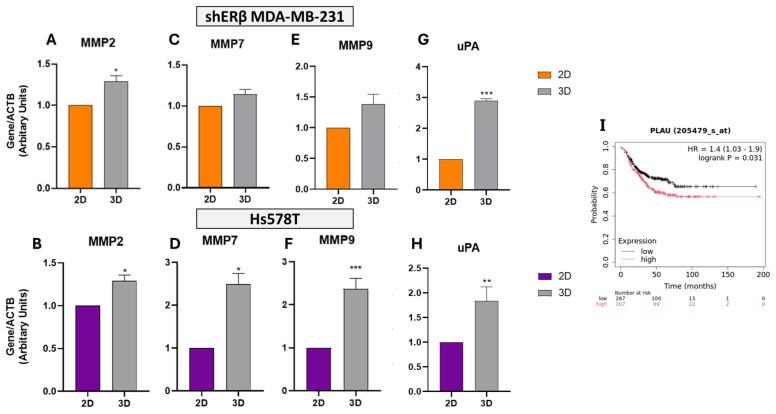
Relative expression of matrix metalloproteinases and uPA in 2D versus 3D cultures of TNBC cell lines. (**A**–**H**) Real-time qPCR analysis of *MMP2*, *MMP7*, *MMP9*, and *PLAU* (*uPA*) in shERβ MDA-MB-231 (orange) and Hs578T (purple) BC cell lines cultured under 2D and 3D conditions. Three-dimensional spheroid culture induced elevated mRNA expression of *MMP2* and *MMP7* in both cell lines, while *MMP9* was robustly upregulated in Hs578T spheroids. *uPA* expression was significantly increased under 3D conditions in both models. Bars indicate mean ± SD from triplicate experiments. Each bar indicates the mean ± SD from triplicate experiments. Asterisks denote statistically significant differences between 2D and 3D conditions: (*) *p*  <  0.05, (**) *p*  <  0.01, and (***) *p*  <  0.001, as determined by Student’s *t*-test. (**I**) Kaplan–Meier survival plot from TNBC patients (KM Plotter) showing that high *PLAU* expression is significantly associated with worse relapse-free survival (HR = 1.4, *p* = 0.031), while *MMP2*, *MMP7*, and *MMP9* showed no significant survival association (*MMP2*: HR = 0.92, *p* = 0.58; *MMP7*: HR = 0.92, *p* = 0.57; and *MMP9*: HR = 1.05, *p* = 0.76).

To further assess ECM remodeling potential under 3D conditions, we analyzed the expression of MMPs in both cell lines. Quantitative RT-PCR revealed differential modulation of *MMP2*, *MMP7*, and *MMP9* in spheroids compared to 2D cultures (Figure 6A–F). In shERβ MDA-MB-231 cells, *MMP2* expression increased by ~1.3-fold (*p* < 0.05), while *MMP7* and *MMP9* showed modest upregulation without reaching statistical significance (Figure 6A,C,E). Baseline transcriptomic values from the HPA for these genes in MDA-MB-231 are minimal (*MMP2*: 0.1 TPM; *MMP7*: 0.3 TPM; and *MMP9*: 0.0 TPM), suggesting low endogenous expression levels.

In contrast, Hs578T spheroids exhibited robust and statistically significant upregulation of all three MMPs under 3D conditions. Specifically, *MMP2* increased by ~1.3-fold (*p* < 0.05), *MMP7* by ~2.6-fold (*p* < 0.05), and *MMP9* by ~2.3-fold (*p* < 0.001) compared to 2D cultures (Figure 6B,D,F). This pronounced induction of *MMP9* aligns with its known involvement in invasive behavior and matrix degradation. Corresponding HPA transcriptomic data further support high basal expression of *MMP2* in Hs578T cells (401.3 TPM), reinforcing its role in ECM remodeling.

However, Kaplan–Meier survival analysis in TNBC patients revealed that high expression of *MMP2*, *MMP7*, or *MMP9* has not been significantly correlated with RFS. Specifically, the survival metrics were as follows: *MMP2*: HR = 0.92, *p* = 0.58; *MMP7*: HR = 0.92, *p* = 0.57; and *MMP9*: HR = 1.05, *p* = 0.76, indicating limited prognostic relevance in this context. Although these enzymes are upregulated in 3D cultures and contribute to ECM degradation, their expression alone does not predict clinical outcome in TNBC cohorts, underscoring the complexity of matrix-driven tumor progression.

To further explore matrix-associated remodeling in 3D culture, we analyzed the expression of *uPA* (*PLAU*) in both BC cell lines. *PLAU* expression was also markedly upregulated in spheroids, exhibiting a ~2.9-fold increase in shERβ cells (*p* < 0.001) and 1.8-fold in Hs578T cells (*p* < 0.01) (Figure 6G,H), consistent with increased proteolytic remodeling activity.

Supporting these findings, transcriptomic data from the HPA showed high baseline expression of *PLAU* (329.3 TPM) in MDA-MB-231 cells, while in Hs578T cells, expression levels were 68.6 TPM, reinforcing its relevance across models.

Kaplan–Meier survival analysis in TNBC cohorts further demonstrated that high expression of PLAU is significantly associated with poorer RFS (HR = 1.4, *p* = 0.031) (Figure 6I), suggesting that 3D-induced upregulation of *PLAU* contributes to an aggressive, matrix-remodeling phenotype linked to unfavorable clinical outcomes in TNBC.

### 3.3. Spheroids Modulate miRNAs—Driven Regulation and Matrix-Mediated Signaling

**Figure 7:**
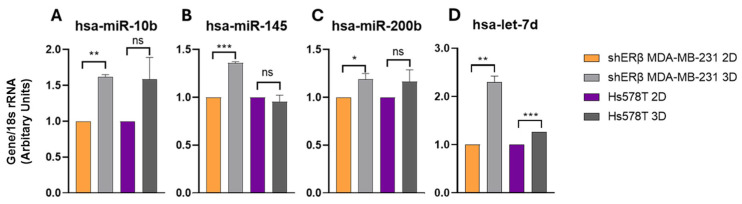
Expression levels of BC-associated microRNAs ((**A**) hsa-miR-10b, (**B**) hsa-miR-145, (**C**) hsa-miR-200b, and (**D**) hsa-let-7d) in 2D and 3D cultures of shERβ MDA-MB-231 and Hs578T BC cell lines. Real-time qPCR was performed to assess relative miRNA expression, normalized to 18srRNA. C2D and C3D represent shERβ MDA-MB-231 cells cultured in 2D and 3D, respectively (orange and light gray bars), while H2D and H3D represent Hs578T cells in 2D and 3D (purple and dark gray bars). Notably, miR-10b, miR-145, miR-200b, and let-7d showed significant regulation in 3D cultures compared to 2D in the shERβ MDA-MB-231 line, whereas no significant differences were observed in the Hs578T line. Each bar indicates the mean ± SD from triplicate experiments. Asterisks denote statistically significant differences between 2D and 3D conditions: (*) *p*  <  0.05, (**) *p*  <  0.01, (***) *p*  <  0.001, and ns (or absent) = not significant as determined by Student’s *t*-test.

To investigate the influence of 3D architecture on post-transcriptional gene regulation, we examined the expression of selected microRNAs (miRNAs) involved in ECM remodeling and BC progression (Figure 7A–D). In shERβ MDA-MB-231 spheroids, hsa-miR-10b expression was increased by ~1.7-fold (*p* < 0.01) and hsa-miR-145 by 1.4-fold (*p* < 0.001) relative to 2D cultures (Figure 7A,B), suggesting that 3D culture induces both pro-invasive and compensatory suppressive miRNA responses. In contrast, no significant changes in these miRNAs were observed in Hs578T cells.

hsa-miR-200b exhibited a modest but non-significant increase (~1.2-fold, *p* > 0.05) in shERβ spheroids, while remaining unchanged in Hs578T (Figure 7C). Notably, hsa-let-7d, an established tumor suppressor miRNA, was significantly upregulated (~2.1-fold, *p* < 0.01) in shERβ 3D cultures, whereas it remained low in Hs578T cells (Figure 7D). These results indicate that 3D spheroid architecture and ERβ status jointly modulate miRNA profiles, with shERβ cells showing more pronounced miRNA responsiveness to 3D-induced cues than Hs578T.

Collectively, the data suggest that the 3D microenvironment reprograms specific miRNAs involved in tumor growth, invasion, and ECM regulation, possibly contributing to distinct phenotypic and signaling outcomes between different TNBC subtypes.

## 4. Discussion

Through this study, we achieved a comprehensive analysis of how BC cells adapt to 3D culture environments, focusing on shERβ MDA-MB-231 cells and the TNBC cell line, Hs578T. By leveraging 3D spheroid models, we addressed limitations inherent to conventional 2D cultures and uncovered key differences in cellular morphology, functionality, and molecular expression profiles. It is noticed that assays performed on a 2D plastic surface, upon transferring the cells from the 3D condition, lack the physiological complexity of a native ECM. However, the obtained results highlight how 3D culture conditions more faithfully recapitulate the TME, influencing cell behavior and gene regulation in a context-specific manner.

Morphological assessments using both phase-contrast microscopy and SEM revealed that spheroid formation was promoted at least a partial MET, aligning with *F11R* expression analysis, with increased compactness and epithelial-like characteristics, particularly evident in Hs578T spheroids. These changes indicate that the 3D architecture induces cellular reprogramming and polarity restoration. The dissemination assay demonstrated the dynamic plasticity of spheroid-derived cells, as both lines exhibited migratory behavior upon reattachment. However, shERβ MDA-MB-231 cells showed greater dispersal capacity, suggesting an enhanced ability of ECM remodeling.

Importantly, spheroid-derived shERβ MDA-MB-231 cells displayed significantly faster wound closure compared to their 2D counterparts, a pattern not observed in Hs578T cells. This lack of enhancement in Hs578T may reflect their intrinsically high baseline migratory capacity, which could mask any further motility gains from 3D conditioning. Moreover, as a mesenchymal-like TNBC subtype with inherently low E-cadherin and high SLUG expression, Hs578T cells may be less responsive to 3D-induced epithelial reprogramming and more matrix-insensitive, relying on constitutive motility programs rather than microenvironmental cues. Such cell line-specific differences in spheroid responsiveness have been reported in other studies, where mesenchymal TNBC models showed limited phenotypic modulation compared to more plastic epithelial-like lines [26,27]. This disparity likely reflects differences in ERβ status, baseline mesenchymal traits, or ECM receptor signaling, reinforcing that 3D-induced phenotypic shifts are cell line-dependent.

The gene expression analysis revealed consistent upregulation of key matrix-related genes under 3D conditions. Both *EGFR* and *F11R* were elevated, indicating activation of receptor-mediated signaling and epithelial cohesion, while MMPs, particularly *MMP7* and *MMP9*, were highly expressed in Hs578T spheroids, pointing to increased proteolytic activity and metastatic potential. *SDC4*, *uPA*, and *IGF1R* were also notably upregulated, implicating them in ECM interaction, degradation, and survival signaling, respectively.

Baseline transcriptomic differences between the two cell lines further contextualized the spheroid-induced responses. Hs578T cells showed inherently high *MMP2* levels, while shERβ MDA-MB-231 exhibited elevated *SDC4* and *uPA* expression, highlighting intrinsic molecular signatures, ultimately shaping cellular behavior in 3D.

Although *MMP2*, *MMP7*, and *MMP9* were markedly upregulated in our 3D culture models—consistent with their known roles in ECM degradation—their elevated expression did not translate into significantly poorer RFS in TNBC patients (*MMP2*: HR = 0.92, *p* = 0.58; *MMP7*: HR = 0.92, *p* = 0.57; and *MMP9*: HR = 1.05, *p* = 0.76). This discordance highlights that MMP expression may serve as a context-dependent modulator of tumor behavior rather than a universal prognostic marker. Indeed, the prognostic value of MMPs varies depending on their cellular origin (tumor vs. stroma) and disease setting, with some studies even reporting anti-tumor roles in certain contexts [28]. Moreover, in TNBC, histological MMP9 expression—particularly in residual post-chemotherapy tumor tissue—has been shown to predict disease-free survival, suggesting a context-specific predictive rather than prognostic role for MMP9 in this subtype [29].

Interestingly, the selective upregulation of miR-10b and miR-145 only in shERβ MDA-MB-231 spheroids—absent in Hs578T—suggests that ERβ status may dictate the extent of transcriptional and post-transcriptional plasticity in response to 3D microenvironment cues. As ERβ is known to regulate these miRNAs inversely—namely suppressing miR-10b and upregulating miR-145—its partial or suppressed presence may preserve the capacity for dynamic miRNA modulation under ECM-rich conditions, promoting adaptive behavior. Conversely, the lack of such responsiveness in ERβ-negative Hs578T cells may reflect a fixed mesenchymal state with reduced sensitivity to microenvironmental signaling. This mechanism aligns with prior findings where ERβ modulation altered miR-10b and miR-145 profiles and downstream EMT/ECM programs in MDA-MB-231 models [21,30].

Finally, miRNA profiling revealed striking differences, particularly in shERβ MDA-MB-231 spheroids, where miR-10b, miR-145, miR-200b, and let-7d were significantly upregulated—miRNAs that regulate EMT, cell migration, and tumor suppression. Their coordinated modulation in 3D conditions suggests a tight interplay between miRNA-driven transcriptional control and matrix-mediated signaling. Notably, miR-200b is recognized for its role in promoting mesenchymal-to-epithelial transition and enhancing chemosensitivity in TNBC—e.g., ARRDC3-mediated upregulation of miR-200b reverses EMT phenotypes and reduces chemoresistance. Broader miRNA-EMT axis dynamics are implicated in therapy resistance across cancers [31,32]. Thus, this miRNA expression pattern may reflect heightened EMT/MET plasticity linked to dynamic adaptation and therapeutic responsiveness. In contrast, Hs578T cells exhibited minimal miRNA changes—suggesting a more fixed mesenchymal phenotype with possible implications for diminished adaptive and drug-response capabilities.

## 5. Conclusions

Collectively, our findings demonstrate that 3D spheroid models significantly influence BC cell behavior by altering their morphology, functional properties, gene expression of matrix effectors, and miRNA profiles in a cell-specific manner. The distinct responses of shERβ MDA-MB-231 and Hs578T cells underscore the importance of ERβ signaling ECM interaction profiles and inherent molecular identity in determining how cells respond to microenvironmental architecture. Our insights support the relevance of incorporating 3D models in BC research to obtain physiologically relevant data, especially for studying tumor progression and therapeutic responsiveness. Beyond fundamental biology, this platform is readily adaptable for high-throughput drug screening, enabling the evaluation of therapy responses in a microenvironmentally relevant context. Moreover, its design permits integration with stromal components—such as cancer-associated fibroblasts (CAFs), macrophages, or other immune cells—to better recapitulate the complexity of the TME and interrogate cell–cell and cell–matrix crosstalk under therapeutic pressure. Although the utilization of standardized in vitro assays on 2D plastic surfaces is useful to evaluate the initial steps of cancer cells-derived 3D spheroids’ spreading and migration, it cannot be considered that reflect a physiological condition since such a model lacks physiological ECM interaction. Future studies should expand upon this framework by incorporating additional TME cell and ECM components—such as fibroblasts, immune cells, and vascular networks, as well as collagen type I and basement membrane macromolecular networks—and integrating proteomic, transcriptomic, and pharmacological profiling. Establishing hybrid models with matrix-based bioscaffolds will bridge the gap between 2D *in vitro* systems and *in vivo* tumor models, providing robust platforms for preclinical cancer research and drug discovery.

## Figures and Tables

**Table 1:** Primer sequences used for quantitative real-time PCR (qPCR). Forward (F) and reverse (R) primers were designed for the detection of target genes associated with extracellular matrix remodeling, growth factor signaling, and cell adhesion in BC. All sequences are listed in the 5′ to 3′ direction. Gene targets include *syndecan-4* (*SDC4*), *epidermal growth factor receptor* (*EGFR*), *insulin-like growth factor receptor* (*IGF1R*), matrix metalloproteinases (*MMP2*, *MMP7*, and *MMP9*), *urokinase-type plasminogen activator* (*uPA*), and *F11 receptor* (*F11R*).

Gene		Primer Sequence (5′→3′)
*SDC4*	F	AGGACGAAGGCAGCTACTCCT
	R	TTTGGTGGGCTTCTGGTAGG
*EGFR*	F	ATGCTCTACAACCCCACCAC
	R	GCCCTTCGCACTTCTTACAC
*IGF1R*	F	ACGAGTGGAGAAATCTGCGG
	R	ATGTGGAGGTAGCCCTCGAT
*MMP2*	F	CGTCTGTCCCAGGATGACATC
	R	ATGTCAGGAGAGGCCCCATA
*MMP7*	F	GCTGGCTCATGCCTTTGC
	R	TCCTCATCGAAGTGAGCATCTC
*MMP9*	F	TTCCAGTACCGAGAGAAAGCCTAT
	R	GGTCACGTAGCCCACTTGGT
*uPA*	F	ACTACTACGGCTCTGAAGTCACCA
	R	GAAGTGTGAGACTCTCGTGTAGAC
*F11R*	F	CCGTCCTTGTAACCCTGATT
	R	CTCCTTCACTTCGGGCACTA
*ACTB*	F	TCAAGATCATTGCTCCTCCTGAG
	R	ACATCTGCTGGAAGGTGGACA

**Table 2:** Mature sequences of selected miRNAs and primer sequences for the normalizer used in qPCR assays. The table lists the mature forms of miRNAs (hsa-miR-10b, hsa-miR-145, hsa-miR-200b, and hsa-let-7d) involved in BC regulation. The 18S rRNA served as the internal reference for normalization of miRNA expression, with forward (F) and reverse (R) primer sequences shown.

**miRNA**		**Mature miRNA Sequence**
hsa-miR-10b-5p		TACCCTGTAGAACCGAATGTG
hsa-miR-145-5p		GTCCAGTTTTCCCAGGAATCCCT
hsa-miR-200b-3p		TAATACTGCCTGGTAATGATGA
hsa-let-7d-5p		AGAGGTAGTAGGTTGCATAGTT
**Normalizer**		**Control Sequence**
18S rRNA	F	CAGGTCTGTGATGCCCTTAGA
	R	GCTTATGACCCGCACTTACTG

## Data Availability

The raw data supporting the conclusions of this article will be made available by the corresponding authors on request.

## Abbreviations

The following abbreviations are used in this manuscript:

2D/3D	Two-/three-dimensional
CAFs	Cancer-associated fibroblasts
DBD	DNA-binding domain
ECM	Extracellular matrix
EGFR	Epidermal growth factor receptor
ERs	Estrogen receptors
ERα/ERβ	Estrogen receptor alpha/estrogen receptor beta
EtOH	Ethanol
FAK	Focal adhesion kinase
HER2	Human epidermal growth factor receptor 2
HMDS	Hexamethyldisilazane
HPA	Human Protein Atlas
IGF1R	Insulin-like growth factor receptor type 1
LOX	Lysyl oxidase
MET	Mesenchymal-to-epithelial transition
MMP	Matrix metalloproteinase
MMP2	Matrix metalloproteinase 2
MMP7	Matrix metalloproteinase 7
MMP9	Matrix metalloproteinase 9
PR	Progesterone receptor
RFS	Relapse-free survival
RT	Room temperature
SD	Standard deviation
SDC4	Syndecan-4
SEM	Scanning electron microscopy
TGF-β	Transforming growth factor beta
TME	Tumor microenvironment
TNBC	Triple-negative breast cancer
uPA	Urokinase-type plasminogen activator
VEGF	Vascular endothelial growth factor
